# Potential Therapeutic Value of the STING Inhibitors

**DOI:** 10.3390/molecules28073127

**Published:** 2023-03-31

**Authors:** Shangran Zhang, Runan Zheng, Yanhong Pan, Hongbin Sun

**Affiliations:** 1 Jiangsu Key Laboratory of Drug Discovery for Metabolic Disease, State Key Laboratory of Natural Medicines, China Pharmaceutical University, Nanjing 210009, China; 2 Department of Pharmacy, The Second Affiliated Hospital of Nanjing Medical University, Nanjing 210009, China; 3 Chongqing Innovation Institute of China Pharmaceutical University, Chongqing 401135, China

**Keywords:** innate immunity, STING, disease, genotype, signal transduction, inhibitors

## Abstract

The stimulator of interferon genes (STING) is a critical protein in the activation of the immune system in response to DNA. It can participate the inflammatory response process by modulating the inflammation-preferred translation program through the STING-PKR-like endoplasmic reticulum kinase (PERK)-eIF2α pathway or by inducing the secretion of type I interferons (IFNs) and a variety of proinflammatory factors through the recruitment of TANK-binding kinase 1 (TBK1) and interferon regulatory factor 3 (IRF3) or the regulation of the nuclear factor kappa-B (NF-κB) pathway. Based on the structure, location, function, genotype, and regulatory mechanism of STING, this review summarizes the potential value of STING inhibitors in the prevention and treatment of infectious diseases, psoriasis, systemic lupus erythematosus, non-alcoholic fatty liver disease, and other inflammatory and autoimmune diseases.

## 1. Introduction

The immune system is a complex defense network covering the whole body. Innate immunity is the first host defensive barrier. It plays a pivotal role in identifying pathogens, removing hidden dangers in the body, and maintaining homeostasis. Type I interferons (IFNs), a kind of cytokines mainly secreted by the activated innate immune system, can further activate immune cells and boost host immunity. Pattern recognition receptors (PRRs) are indispensable to innate immune cells and can recognize pathogen-associated molecular patterns (PAMPs) and damage-associated molecular patterns (DAMPs) [1]; upon detection by PRRs, PAMPs, or DAMPs, they activate proinflammatory signaling pathways, release inflammatory factors, and initiate adaptive immunity. PRRs signaling can be regulated by multiple pathways in order to eliminate microorganisms promptly and maintain the proper immune response to avoid adverse effects [2]. Regardless of stimulus, excessive immune system activation can lead to various inflammatory and autoimmune diseases [3]. Currently, studies have reported that three types of PRRs participate in the recognition of pathogenic nucleic acids and the induction of type I IFN secretion, namely toll-like receptors (TLRs) [4], RIG-I-like receptors (RLRs) [5,6,7], and DNA recognition receptors [8,9].

STING is an adjuvant protein on the endoplasmic reticulum (ER) which recognizes the cyclic dinucleotides (CDNs) generated by cyclic GMP-AMP synthase (cGAS) [10]. cGAS can recognize not only foreign DNA, but also DNA from the cell itself [11]. Once cGAS senses cytoplasmic double-stranded DNA (dsDNA), it converts GTP and ATP into 2′,3′-cGAMP, which binds and activates STING [12], and it eventually secretes type I IFN and various proinflammatory factors [13]. As part of the feedback loop of IFNs, both cGAS and STING expression can be upregulated positively by type I IFNs in order to amplify the immune response [14,15]. Meanwhile, STING and its upstream IFN-γ inducible protein 16 (IFI16) are also downregulated in order to prevent excessive inflammation [16,17]. The over-activation of STING is reported to be related to psoriasis, systemic lupus erythematosus (SLE), infectious diseases, non-alcoholic fatty liver disease (NAFLD), and other interferonopathies including STING-associated vasculopathy in infants (SAVI) and Aicardi–Goutières syndrome (AGS) [13,18,19,20]. Many STING inhibitors have been discovered, and their activities have been continuously improved through structural modifications. Li et al. isolated a STING inhibitor—Astin C—from the natural plant *Aster tataricus*, which was reported to alleviate palmitic acid-induced cardiomyocyte contractile dysfunction [21,22]. C-176, the covalent STING inhibitor, showed remarkable efficacy in mouse models of diabetic cardiomyopathy and regulated pancreatic β-cell function [23,24]. Moreover, H-151, which was obtained through high-throughput screening after C-176, was tested with promising results in treating psoriasis [25], myocardial infarction [26], acute kidney injury [27], and acute lung injury in mice [28]. However, due to its relatively weak potency and poor pharmacokinetic properties, current STING inhibitors are still in the early stages, and there are no compounds being tested in clinical studies. Moreover, the high heterogeneity of the human STING (hSTING) gene also presents challenges to the development of STING inhibitors with clinical potential [29].

In this article, we summarize the current progress in research on STING, focusing on the structure, function, genotype, regulatory mechanism, current inhibitors, and therapeutic potentials of STING inhibitors in treating inflammatory and autoimmune diseases.

## 2. The Structure and Location of STING

STING (also known as TMEM173, MITA, ERIS, and MPYS) [30,31,32,33,34] is an ER membrane protein composed of 379 amino acids [35]. The C-terminus domain (CTD) of STING contains a ligand-binding domain (LBD), and the N-terminus contains four transmembrane (TM) domains. The C-terminus also includes a dimerization domain and a C-terminal tail (CTT) domain containing the phosphorylation site of TANK-binding kinase 1 (TBK1) [36]. The dimerization domain consists of hydrophobic amino acids that approach each other through hydrophobic interactions to form a V-shaped dimer where the binding site for cyclic dinucleotides (CDNs) is located [37,38,39]. CDNs include cyclic diadenosine monophosphate (c-di-AMP), cyclic diguanylate (c-di-GMP), and cyclic GMP-AMPP (cGAMP). In mammalian cells, the endogenous cGAMP includes two phosphodiester linkages: one between GMP 2′-OH and AMP 5′-phosphate and the other between AMP 3′-OH and GMP 5′-phosphate, which is called 2′,3′-cGAMP [40,41]. Compared with alternatively formed cGAMPs, 2′,3′-cGAMP showed the strongest affinity to hSTING, with a reported K_d_ value 300 times lower than that of c-di-GMP and 3′,3′-cGAMP, and 75 times lower than 2′,2′-cGAMP [21,40]. Additionally, the binding of c-di-GMP to STING is exothermic, while that of 2′,3′-cGAMP is endothermic [40].

Based on the Human Protein Atlas (HPA) database [42], STING expression has low tissue specificity and is expressed in almost all tissues. Except for B cells, STING is expressed in almost all immune cells. STING expression is higher in the lung, bronchi, tonsil, lymph node, and spleen, and lower in the skin, stomach, kidney, and adipose tissue.

## 3. The Function of STING

**Figure 1:**
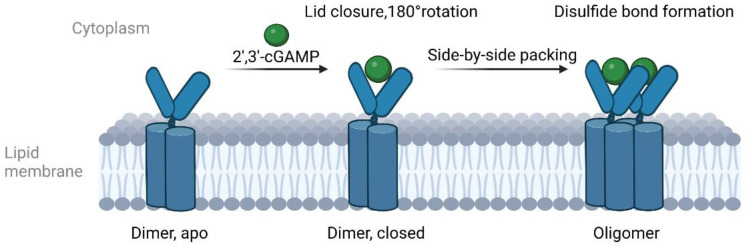
Cartoon model of the cGAMP-induced oligomerization of wild-type STING.

The cytoplasm of eukaryotic cells is devoid of DNA under normal physiological conditions, and any small amounts that may leak into the cytoplasm are swiftly degraded by nucleases [43]. The detection of cytoplasmic DNA by the innate immune system induces various inflammatory responses and defense mechanisms [39]. Since there is no DNA-binding domain in the STING structure, other pattern-recognition receptors are required in order to help recognize DNA, like cGAS. The threshold of STING activation is vital for the body to distinguish its own basic DNA level from abnormal conditions [44]. Under unstimulated conditions, STING predominantly resides on the ER, although it has also been reported to be present on the mitochondria-associated ER membrane [30]. Upon activation, STING traffics via the Golgi apparatus to form discrete punctate polymers around the nucleus [30,45]. DNA receptor cGAS can recognize abnormally exposed cytoplasmic DNA and synthesizes 2′,3′-cGAMP, including viral and bacterial DNA, DNA produced by the reverse transcription of RNA viruses, and DNA produced by cell damage [45]. The binding of cGAMP to the STING dimer induces the rotation and closure of STING LBD and the release of STING CTT, leading to the formation of disulfide-linked polymers (Figure 1) [44,46]. STING oligomerization occurs in the ER [44]. After activation, STING is translocated from the ER via the ER–Golgi intermediate compartment (ERGIC) to the Golgi apparatus, where TBK1 and interferon regulatory factor 3 (IRF3) are recruited [36,47]. TBK1 phosphorylates STING and IRF3 after TBK1 autophosphorylation, inducing the dimerization of IRF3 [36,47]. IRF3 dimers are then transported into the nucleus and promote the expression of type I IFN and related immune factors. After activation, STING is transported to the lysosome for degradation [48,49].

The expression of inflammatory factors can be upregulated by activation of the NF-κB pathway [50]. TRAF6, an E3 ubiquitin ligase of the TRAF family, is essential in the non-canonical NF-κB pathway during STING activation [51,52,53,54]. Abe et al. reported that STING and TBK1 could facilitate the dsDNA-induced activation of the NF-κB pathway with the assistance of TRAF6 [52]. Additionally, Ataxia Telangiectasia mutated protein (ATM) and poly(ADP-ribose) polymerase 1 (PARP-1) can form a STING-activating complex with TRAF6, IFN-γ inducible protein 16 (IFI16), and p53 in response to DNA damage [53]. Subsequently, TRAF6 catalyzes STING K63-linked ubiquitination in order to activate the NF-κB pathway independently of TBK1 [53]. The special sequence of the zebrafish STING CTT can even recruit TRAF6 in order to boost NF-κB signaling [54]. In contrast, TRAF6 and TBK1 play dispensable roles in the canonical NF-κB response induced by STING [55]. These findings shed light on the intricate and diverse mechanisms underlying STING-mediated immune responses.

Recently, a novel discovery highlighted the STING-PKR-like ER kinase (PERK)-eukaryotic initiation factor-2α(eIF2α) pathway independently of the classical STING cascades [56]. Upon cGAMP activation, STING was able to bind to and activate PERK, promoting PERK-mediated eIF2α phosphorylation [56]. Through this pathway, DNA damage suppresses cap-dependent mRNA translation and turns cells to inflammation- and survival-biased translation programs, contributing to organ fibrosis and cellular senescence [56].

Autophagy is a highly conserved intracellular degradation process used to remove damaged organelles, protein aggregates, and invading pathogens [57]. Studies have shown that STING-mediated autophagy is required for innate immunity and the termination of cGAS-STING signaling [50,58]. Structurally, STING includes the light chain-3 (LC-3)-interacting regions (LIRs) through which STING can directly interact with LC-3 in order to regulate autophagy [59]. Upon cGAMP-induced activation and dimerization, STING is translocated to ERGIC and binds to LC-3 and WD repeat domain, phosphoinositide interacting 2 (WIPI2) [59,60]. STING-containing ERGIC facilitates LC3 lipidation and the assembly of autophagosome depending on autophagy-related 5 (ATG5) and WIPI2 [59,61]. During this process, TBK1 and UNC-51-like kinase 1 (ULK1/ATG1), which were found to downregulate STING, are dispensable [59,61,62].

## 4. Genotype

**Figure 2:**
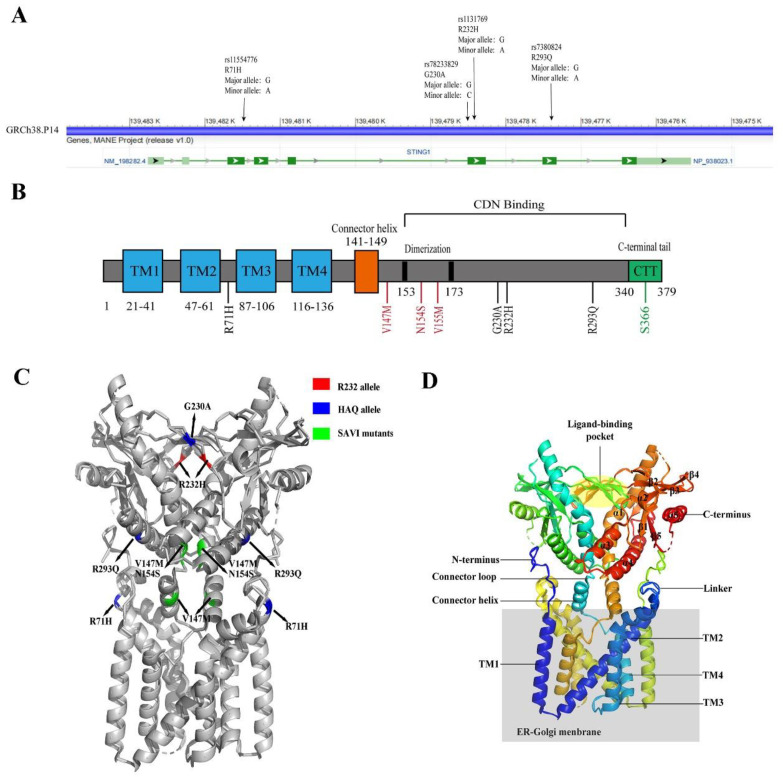
The structure of hSTING. (**A**) The hSTING gene map (NCBI reference sequence: NC-000005.10), annotated with common SNPs. (**B**) Schematic diagram of the hSTING protein domain. Transmembrane domains are marked in blue, common human mutation points are marked in black, regions related to IRF3 activation are marked in green, and SAVI mutation points are marked in red. (**C**) The crystal structure of the hSTING protein (PDB: 7SII). R232 is marked in red, the HAQ mutation point is marked in blue, and the SAVI mutation point is marked in green. (**D**) Representation of the STING structure (PDB: 7SII). The secondary protein structure, N-terminus, and C-terminus are numbered. The ER–Golgi membrane is colored in gray.

There is 81% structural similarity between hSTING and murine STING (mSTING) [63], but hSTING has obvious heterogeneity. STING R232, not H232, is the most common hSTING allele, accounting for about 45% of the population [64,65]. Seema et al. found that R71H-G230A-R293Q (HAQ) was the second most-common hSTING allele (Figure 2), accounting for about 16.07% of East Asians, about 7.78% of South Americans, and 6.75% of South Asians [65]. R232/R232 is the predominant STING genotype of Europeans, and R232/HAQ is the most common hSTING genotype among East Asians [65]. Approximately 10% of Europeans and 31% of East Asians have HAQ/HAQ, HAQ/H232, and H232/H232 genotypes [65]. G230A and R232H are localized on the top of STING binding pocket, and R293Q is located at the bottom [66]. R71H is located at a cytoplasmic loop facing the bottom, and SAVI mutants are located in the stem region of the binding pocket (Figure 2) [66].

A recent study suggested that HAQ and H232 might be STING loss-of-function alleles [65]. Neither IRF3 nuclear translocation nor IFN-β production was observed in B cells that were homozygous H232 or HAQ/H232 stimulated with 2′,3′-cGAMP cyclic di-AMP (CDA), cyclic di-GMP (CDG), or RpRp-ssCDA [65]. Although the activity of STING HAQ was greatly reduced, this did not translate into a loss of function [67]. STING HAQ presented a more than 90% loss in its ability to stimulate IFN-β secretion due to R71H and R293Q in HAQ [64]. The C292 and C88xxC91 near R293Q and R71H also played important roles in IFN-β stimulation [64]. Compared with HAQ-expressing cells, the Q293-expressing cells displayed a mild deficiency of IFN-β stimulation [64]. Furthermore, the H71 allele resulted in a more-severe deficiency in IFN-β stimulation than the Q293 allele [64]. However, the H71 allele alone stimulated IFN-β secretion more effectively than HAQ [64]. The defective IFN-β production capacity in HAQ carriers may be associated with the low level of STING protein expression and TMEM173 transcription. In addition, H232 has low binding affinity with CDNs [65]. The STING expression in H232/H232 B cells is comparable to that in R232/R232 cells, while it is reduced in HAQ/H232 because of the HAQ allele [65].

It has been reported that SAVI patients with HAQ had SAVI symptoms, but the onset of the disease was delayed (by approximately 3 years) [68]. STING HAQ is associated with increased susceptibility to Legionnaires’ disease. Compared with the healthy people, the proportion of STING HAQ was raised in two groups of Legionnaires’ disease patients, while STING R232H was not increased [67]. Although the replication of *Legionella pneumophila* in wild-type (WT) STING and HAQ-type cells are similar, the production of IFN-β and IP-10 was reduced in homozygous HAQ cells, which were stimulated with cGAMP, *Legionella* infection, synthetic DNA, and bacterial DNA, and this effect did not occur in response to the TLR7/8 agonist Resiquimod [67]. Furthermore, the expression of IFN-β and IL-1β of heterozygous HAQ carriers was partially decreased [67]. Therefore, the differences in STING expression and function found in individuals with HAQ allele may affect the therapeutic effect of STING-target treatment.

## 5. DNA Sensors Upstream of STING

Besides cGAS, DEAD-box RNA helicase 41 gene (DDX41) and IFI16 have been confirmed to participate in DNA recognition and get involved in STING activation [69,70,71,72].

The cGAS C-terminal domain includes a unique zinc band vital to binding to DNA [73]. cGAS can bind to DNA pentose phosphate backbone and form a 2:2 cGAS-DNA complex in order to produce cGAMP and activate STING, thereby releasing IFN-β [74]. There are effects of the type and length of nucleic acids bound to cGAS and their status on cGAS activity. DNA base oxidation induced by UV irradiation cannot affect the binding ability with cGAS [75]. Single-stranded DNA (ssDNA) and double-stranded RNA (dsRNA) binding to cGAS cannot rearrange the structure of the catalytic pocket and thus cannot induce the production of cGAMP [72,75,76]. The cGAS-STING pathway preferentially responds to long fragments of cytosolic DNA (>45 bp), which is related to cGAS K187 and L195 [77]. Compared with murine cGAS, human cGAS K187 and L195 mutations result in increased sensitivity to long DNA fragments and decreased sensitivity to short DNA fragments [77]. cGAS activity is also affected by post-translational modifications and the ionic environment. Mn^2+^ can improve the sensitivity and activity of cGAS towards dsDNA while increasing the binding affinity of STING to cGAMP [78]. Zn^2+^ can improve recombinant cGAS activity in the buffer, which can be partially replaced by Mn^2+^ and Co^2+^ [76].

IFI16 is a member of the PYRIN and HIN200 domain-containing (PYHIN) protein family [79]. p204 is the mouse PYHIN most similar to IFI16, containing a pyrin domain and two DNA-binding HIN domains, which can directly bind to viral dsDNA to produce IFN-β [71]. IFI16/p204 is considered to be the first PYHIN protein involved in IFN-β production and cellular senescence [71,80]. As the DNA-sensing protein, IFI16/p204 cooperates with cGAS in cGAMP-induced STING activation to defend from viral infection [81,82]. Under normal conditions, IFI16 is located in the cell nucleus [83]. Upon the recognition of viral DNA, IFI16 polymerizes, forms an inflammasome complex, and is transported to the cytoplasm [71,84]. In the cytoplasm, IFI16 recruits TBK1 to STING and interacts directly with STING through the PYRIN domain, thereby initiating STING-IRF3 activation and promoting IFN-β production [71,82,84]. In turn, STING and IFI16 can also regulate the one another’s protein levels in order to avoid continuous activation [16,17]. Furthermore, IFI16 can detect nuclear DNA damage and participate in the activation of the STING-NF-κB pathway with ATM independently of cGAS [53].

DDX41, a helicase belonging to the DEXDc family, is the DNA sensor of myeloid dendritic cells (mDCs) [69]. DNA can bind directly to the DDX41 DEAD domain, while the STING TM2, TM3, and TM4 domains can interact with DDX41 [69]. Moreover, Walker A and B sequences of DEAD play indispensable roles in interacting with dsDNA or STING [69,84]. DDX41 can recognize dsDNA and interact with the STING-TBK1 complex to regulate IRF3 activation [84]. Furthermore, DDX41 can interact with bacterial c-di-GMP and cGAMP to regulate the type I IFN signaling [84,85].

## 6. The Regulation of the STING Pathway

STING CTT has a self-inhibition effect on STING [44]. The recruitment of TBK1 alone is insufficient to activate IRF3. STING CTT S366 is a vital site for IRF3 binding and activation [36]. TBK1 can trigger the phosphorylation of STING CTT S366, providing a platform for IRF3 recruitment and TBK1 autophosphorylation. When S366 is mutated, IRF3 activation is blocked [86]. Although STING CTT is indispensable for the STING-TBK1-IRF3 pathway, it is not vital for activating the STING-NF-κB pathway. The dmSTING (amino acids 147–343) of Drosophila lacks CTT but retains the ability to activate the NF-κB pathway through transcription factor Relish [87].

Upon dsDNA stimulation, STING colocalizes with autophagy-related gene 9a (ATG9A) and LC-3 and conducts membrane trafficking in an ATG9A-dependent manner [88]. ATG9A restricts innate immune responses by disrupting the binding between STING and TBK1 [88].

After TBK1 activation, STING induces autophagy in order to degrade itself and p-TBK1 [59]. At activation, STING can form a complex with adaptor protein complex 1 (AP-1) at the Golgi through the CTT domain, especially phosphorylated S366, controlling the degradation of STING through the endolysosomal system [48]. STING S366 can also be phosphorylated by ULK1/ATG1, inducing STING degradation and preventing sustained activation by autophagy [62]. Additionally, STING residues 330–334 play significant roles in autophagy induction. Mutations of L333A and R334A can inhibit LC-3 esterification, TBK1 and IRF3 phosphorylation, and cGAMP-induced STING and LC-3 puncta formation [61]. Unc-93 homolog B1 (UNC93B1) is also able to interact with STING, reducing STING’s stability by delivering STING to the lysosomes for degradation [89,90].

Moreover, human epidermal growth factor receptor 2 (HER2) directly phosphorylates TBK1 by recruiting AKT serine/threonine kinase 1 (AKT1) and hinders the STING-TBK1 interaction and TBK1 K63-linked ubiquitination, attenuating the STING signaling pathway [91,92]. Likewise, when STING C148 was mutated, the affinity of STING to cGAMP was decreased, and the downstream pathway was inhibited [44].

**Figure 3:**
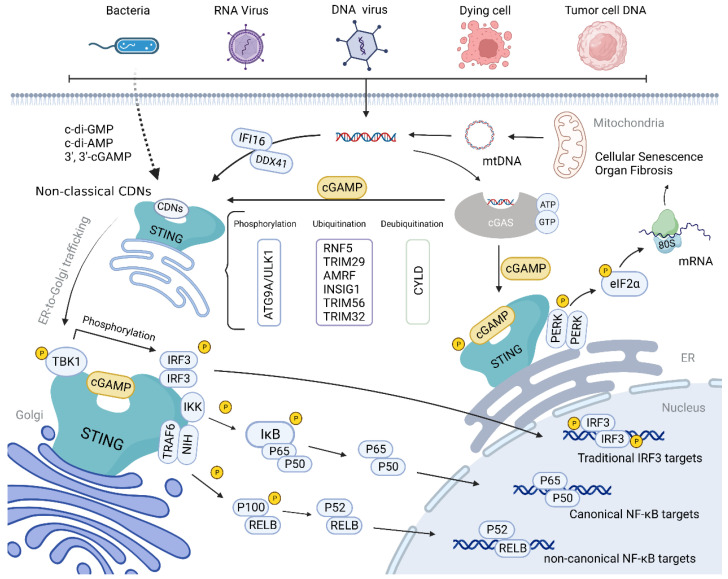
Schematic diagram of the cGAS-STING signaling pathway. cGAS can recognize abnormally exposed cytoplasmic DNA molecules, including viral and bacterial DNA, DNA produced by the reverse transcription of RNA viruses, and DNA produced by self-cell damage. It can catalyze the synthesis of 2′, 3′-cGAMP, which specifically binds to STING dimer for oligomerization. After activation, STING is translocated to the Golgi via ERGIC, during which TBK1 and IRF3 are recruited, and this complex induces an immune response by phosphorylating IRF3 or NF-κB. In addition, STING can activate PERK and promote the phosphorylation of eIF2α, inducing translation program transformation. Autophagy, ubiquitination, recruitment inhibition, mutation, and other pathways can affect the STING pathway.

Within the ubiquitin–proteasome pathway, host cells can degrade STING-pathway inhibitor factors in order to activate the pathway while limiting the continuous activation of the pathway so as to avoid self-injury caused by excessive inflammatory responses (Figure 3) [93]. Autocrine motility factor receptor (AMFR) and insulin-induced genes 1 (INSIG1) are ER-protein and can form a complex which functions as E3 ubiquitin ligase [94]. An AMFR and INSIG1 complex has been reported to catalyze STING K27-linked polyubiquitination [94]. Upon this modification, TBK1 is recruited, inducing STING transfer to the perinuclear body and enhancing STING pathway activation [94]. Contradictorily, ER-localized E3 ubiquitin ring finger protein 5 (RNF5) and tripartite motif protein family member 29 (TRIM29) are able to catalyze the K48-linked ubiquitination of STING K150 and K370, respectively [33,95], resulting in the degradation of STING. Moreover, cylindromatosis (CYLD), a deubiquitinating enzyme, can specifically remove K48-linked ubiquitination, stabilizing the STING protein [96]. Tetsuo et al. found that STING K150 was not only the site of the K48-linked ubiquitination of STING but also an essential amino acid residue for K63-linked ubiquitination [97]. TRIM56 catalyzes K63-linked ubiquitination of Lys150, while TRIM32 catalyzes K63-linked ubiquitination of K20/150/224/236, respectively, promoting STING dimerization and interaction with TBK1 [97,98]. Additionally, it was found that blocking post-translational modifications of STING [99], like ubiquitination at K224 and K228, only inhibited the STING-IRF3 pathway without affecting NF-κB-related responses [100,101].

## 7. STING Inhibitors

The over-release of type I IFN is a significant trigger of numerous IFN-related diseases, including SLE, AGS, and SAVI. Down-regulating the over-activated STING pathway can reduce the type I IFN release, help the immune system return to normal, and maintain the body’s dynamic balance. However, research on STING inhibitors is in the early stages, and no compounds have yet entered clinical trials (Table 1).

### 7.1. STING Inhibitors Targeting the Palmitoylation Site

**Figure 4:**
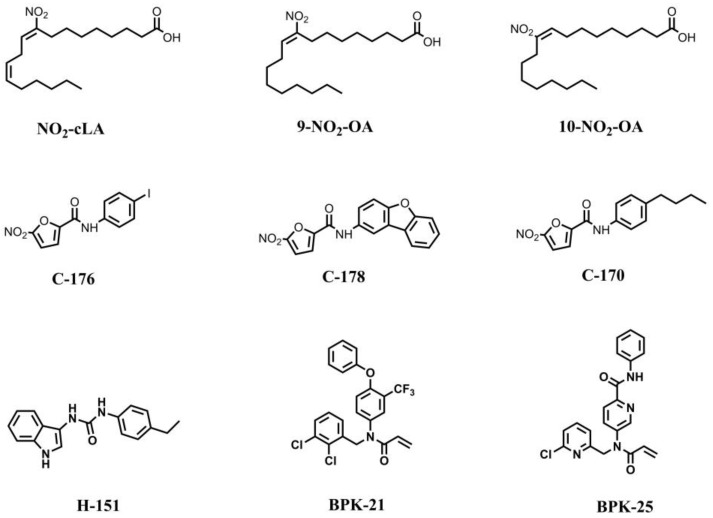
STING inhibitors targeting palmitoylation sites.

Palmitoylation at cysteines 88 and 91 (C88/91) is vital to STING activation [110]. H-151, C-178, C-176, and NO_2_-FAs are the inhibitors targeting the palmitoylation site (Figure 4) [102,103]. The NO_2_-FAs were formed by adding NO_2_ to unsaturated fatty acids during viral infection, including nitro-conjugated linoleic acid (NO_2_-cLA) and nitro-oleic acid (NO_2_-OA) [111,112]. Endogenous NO_2_-FAs covalently modify STING via nitro-alkylating of C88, C91 and H16, thus inhibiting STING palmitoylation and activation, reducing the release of type I IFN [102,113]. Additionally, the Phase II trial of NO_2_-FAs for focal segmental glomerulosclerosis (clinicaltrials.gov: NCT03422510) has been completed, but the Phase II trial for pulmonary arterial hypertension (clinicaltrials.gov: NCT03422510) was terminated, indicating that more validation is needed.

C-176 and C-178 are nitrofuran derivatives whose four-position of the furan ring can undergo a nucleophilic addition reaction with STING C91 in order to form a covalent bond, leading to intramolecular rearrangement and the destruction of the palmitoylation of STING [103]. C-176 and C-178 are able to covalently modify the C91 of mSTING and inhibit its activation, but they fail to block hSTING [103]. Therefore, researchers modified the substituent on the benzene ring in order to obtain C-170, which has an inhibitory effect on both hSTING and mSTING [103]. Additionally, they identified H-151 through high-throughput screening, which suppresses STING palmitoylation and block TBK1 phosphorylation with higher specificity for hSTING than mSTING [103].

Vinogradova et al. identified a series of compounds named BPK as STING inhibitors that reduced cGAMP-induced STING activation [104]. It was proven in silico that the binding sites of these compounds are associated with STING C91, the same as C-178 [1,104]. However, it should be noted that BPK-21 and BPK-25 have limited specificity and can also interact with the cysteine residues of other immune-related proteins.

### 7.2. STING Inhibitors Targeting CDN Binding Site

**Figure 5:**
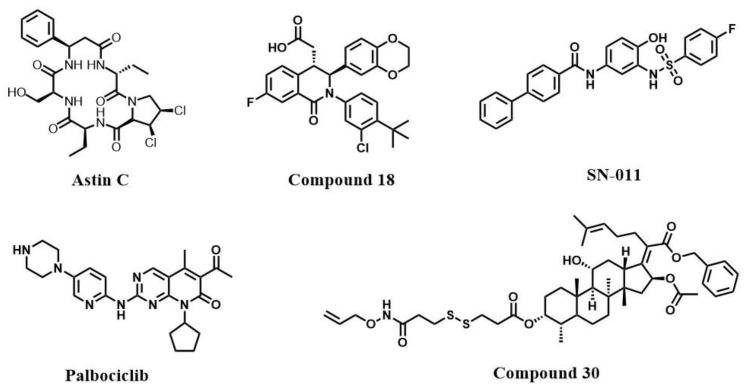
STING inhibitors targeting CDN binding sites.

c-di-GMP can competitively inhibit cGAMP-induced STING activation, and the STING conformation caused by c-di-GMP differs from that of cGAMP [44]. Therefore, any compound which induces conformational changes of STING different from those included by cGAMP may be a potential STING inhibitor (Figure 5).

The small molecule cyclic peptide Astin C is able to bind to the STING C-terminus, block IRF3 recruitment, and inhibit the activation of the STING pathway [21]. However, the polarity and solubility of this compound are both poor, and higher-purity cyclic peptides are difficult to obtain, hindering subsequent research.

The analog of tetrahydroisoquinoline, compound **18**, is a cGAMP competitive inhibitor [105]. Compound **18** produces STING inhibition by competing with cGAMP at a 2:1 ratio for the binding site of inactive conformation [105]. However, this compound has very poor inhibitory activity against hSTING at the cellular level.

We recently reported a novel STING inhibitor, SN-011, which could compete with cGAMP for CDN binding sites and maintain an inactive conformation [106]. SN-011 has been shown to effectively suppress systemic inflammation in Trex1^−/−^ mice with lower cytotoxicity and higher specificity in comparison to H-151 [106].

Gao et al. identified the cyclin-dependent kinase (CDK) inhibitor palbociclib as a STING inhibitor [107]. Palbociclib can interact directly with the STING Y167 to block STING dimerization and alleviate autoimmune disease features.

Recently, Long et al. synthesized a series of fusidic acid derivatives inhibiting the abnormal activation of STING-related pathways [108]. Based on molecular docking, compound **30** can interact directly with the residues of STING CTD, such as T263, G166, and R23.

### 7.3. STING Inhibitor with an Unknown Site of Action

**Figure 6:**
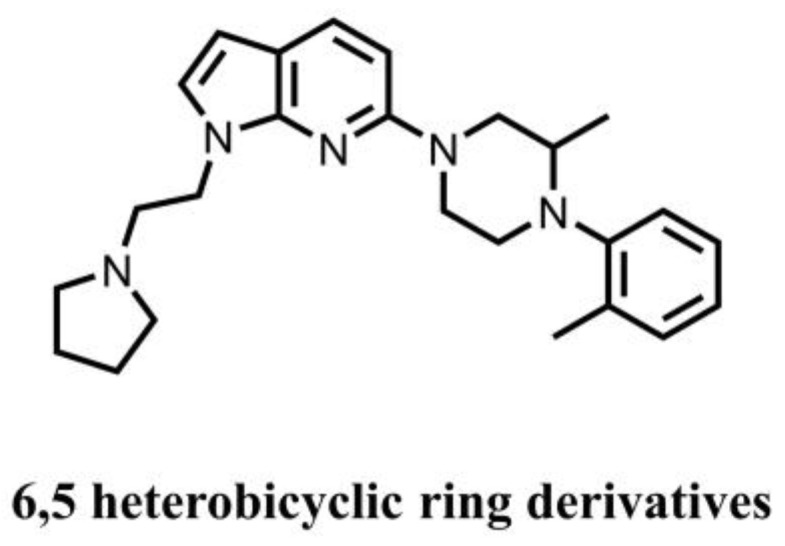
STING inhibitor with an unknown site of action.

Patents EP 3527209 A1 and WO 2019158731 A1 reported that 6,5-heterocyclic derivatives (Figure 6) could reduce the cGAMP-induced secretion of IFNs in HEK-Blue ISG cells. The IC50 values of these compounds range from 30 μM to less than 10 nM. However, the inhibitory effect of these compounds is only directly confirmed by the ISG54 reporter gene detection method and needs further research in order to confirm whether it is a direct STING inhibitor.

### 7.4. STING Protein Degraders

**Figure 7:**
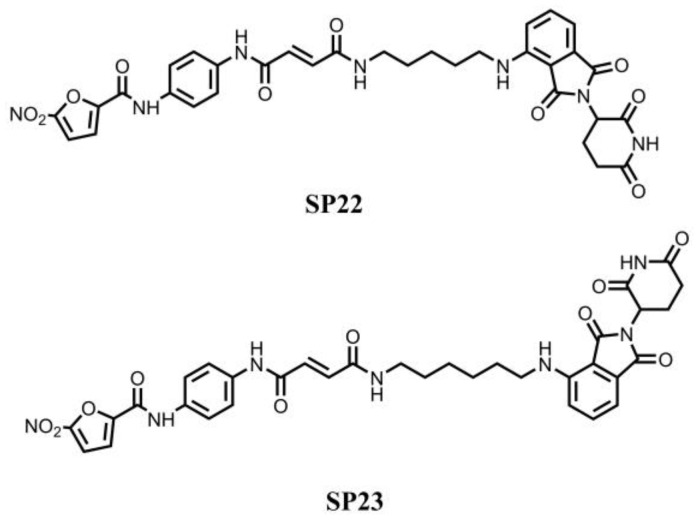
STING protein degraders.

In 2022, Liu et al. used proteolysis-targeting chimera (PROTAC) technology to find a series of STING protein degraders (Figure 7) based on the C-170 and the cereblon (CRBN) ligand pomalidomide [109]. Among these compounds, SP22 and SP23 were able to induce STING degradation in THP1 cells with DC_50_ of 9.2 μM and 3.2 μM, respectively. Furthermore, SP23 exerted an anti-inflammatory effect by modulating the STING pathway in a murine model of acute kidney injury [109]. This is the first example of STING protein degraders.

## 8. STING-Related Diseases

### 8.1. STING and Psoriasis

The inflammatory skin condition psoriasis is recurring, systemic, and chronic and is characterized by aberrant keratinocyte proliferation and inflammatory cell infiltration [114]. The global incidence of this condition rate is about 2–3%, and there are about 6.5 million patients in China, mainly in young and middle-aged people [114,115]. The clinical manifestations of psoriasis are various, and psoriasis vulgaris is the most common, with characteristics including pruritus, scattered red rash, and silvery-white scales scattered over the surface of the skin [116]. The course of this disease is long, and it is difficult to cure. Genes, injury, infection, medicine, living habits, living environment, and other factors can lead to the occurrence and development of psoriasis [117,118].

In 1994, Krueger’s team found that targeting immune cells could alleviate the disease, and confirmed that inflammation was not a side effect [119]. The various functions of keratinocytes and immune cells are being discussed for the first time. Although the pathogenesis of psoriasis is still unclear, the immune system should be crucial in it.

Clinically, the use of type I IFN to treat viral infections often directly induces or aggravates the occurrence of psoriasis [119,120]. Keratinocytes account for 90% of the total number of epidermal cells. Bielenberg et al. reported that keratinocytes could secrete IFN-β and regulate self-differentiation [121]. When skin keratinocytes are damaged, plasmacytoid dendritic cells (pDCs) produce IFN-α/β through the TLR7/8-MyD88-IRF7 pathway. IFN-α/β induces the maturation of mDCs and the secretion of TNF-α, IL-12, and IL-23, activating Th1 and Th17 cells and producing IL-17A and IL-22 [114,122,123]. Meanwhile, T cells are activated through antigen presentation [114,123,124]. Although human keratinocytes are tolerant to intracellular DNA, keratinocytes can acquire the ability to respond to DNA through the antimicrobial peptide LL37 pathway in the presence of inflammatory factors [70]. Therefore, keratinocytes lacking TLR7/8 can secrete IFN-β through the STING pathway to promote inflammation due to the leakage of intracellular DNA and the presence of inflammatory factors [125]. Etoposide-induced DNA damage has a non-classical STING-activation mechanism independent of cGAS [25]. Within a few hours, keratinocytes and other cells exhibit the innate immune response [126]. Given that STING is expressed in almost all tissues, the overproduction of inflammatory factors may exacerbate the over-activation of the STING pathway, creating a vicious cycle. In line with our worries, individuals with moderate to severe psoriasis have heightened proinflammatory factors, not just in their skin but also in their blood [127]. The increased expression of these inflammatory factors increases the risk of developing inflammation-related diseases and various complications, such as psoriatic arthritis, diabetes, and NAFLD [128]. Therefore, the downregulation of proinflammatory factors released through over-activation of the immune system, such as type I IFN, should be beneficial to slowing down the deterioration of psoriasis and preventing complications from occurring.

Currently, the treatment of psoriasis includes local therapy, systemic therapy, and phototherapy, such as UV-B phototherapy. Methotrexate, tretinoin, and calcipotriol have often been used medications for psoriasis. Chinese Food and Drug Administration data show that domestic psoriasis drugs are mostly used topically [129,130]. In recent years, monoclonal antibodies have been introduced to treat moderate to severe psoriasis, and global sales totaled to about 7.49 billion US dollars in 2014 [25,131,132]. Although monoclonal antibodies including TNF-α, IL-23, and IL-17 inhibitors have shown excellent therapeutic effects in clinical practice [133], there are still some problems. Long-term use of TNF-α inhibitors shows side effects such as the reactivation of hepatitis B and C [134], while IL-23 inhibitors carry a risk of nasopharyngitis and upper respiratory tract infections [135]. IL-17 inhibitors, such as Secukinumab, Ixekizumab, and Brodalumab, may induce depression and suicide with long-term medication [136]. Moreover, their prices are so high that some patients are unable to afford them. Therefore, psoriasis patients urgently need a drug with low cost, high efficacy, minimal side effects, and ease of use. Small-molecule drugs may represent a breakthrough, and several are already in clinical trials.

STING can associate innate immunity and adaptive immunity via inflammatory factors such as IFN-β [124]. Recently, we reported that the STING inhibitor H-151 alleviated IMQ-induced psoriatic dermatitis by inhibiting the STING-NF-κB pathway of keratinocytes and macrophages and reducing the secretion of inflammatory factors such as IL-6 and TNF-α [25,137]. Another STING inhibitor, C-176, has also been found to ameliorate the development of psoriasis in diabetes via the STING-IRF3 pathway [138]. Therefore, it might represent a novel treatment for psoriasis and its associated complications through the inhibition of STING [138].

### 8.2. STING and Systemic Lupus Erythematosus

Systemic lupus erythematosus is an autoimmune disease that may involve multiple organs, including the skin, bone marrow, kidneys, joints, muscles, lungs, cardiovascular system, and central nervous system [139,140]. Its serological feature is a high titer of autoantibodies against dsDNA and other nuclear components [19].

Overproduction or poor clearance of neutrophil extracellular traps (NETs), including nuclear histone, depolymerized chromatin, various granule proteins, and certain cytoplasmic proteins, may be associated with SLE [141]. The anti-DNase antibody, which is positive in 62% of SLE patients, can protect NETs from degradation by binding to a conserved region near the DNase catalytic site [142]. The neutrophil antimicrobial peptide LL37 and dsDNA in the serum of SLE patients are able to form immune complexes (ICs) as autoantigens, which can in turn activate B cells through B-cell receptors and TLR9 in order to produce anti-DNA and anti-LL37 antibodies [143].

Despite the high heterogeneity of SLE, more than 80% of patients experienced altered IFN signaling [144]. Clinically, SLE can occasionally develop following recombinant IFN-α therapy for chronic hepatitis [19]. The levels of dsDNA and type I IFN are upregulated in SLE patients, and the expression of type I IFN-stimulating genes (ISGs) in peripheral blood mononuclear cells is higher in SLE patients than in those with other autoimmune diseases or healthy patients [145,146]. Apoptosis-derived membrane vesicles in the serum of SLE patients have high ISG-inducing activity, and the knockout of cGAS or STING reduces this activity [146]. Gkirtzimanaki et al. demonstrated that excessive IFN-α disrupts mitochondrial metabolism, inducing oxidative stress, impaired lysosomal degradation, and autophagy obstruction [147]. The accumulation of cytosolic mitochondrial DNA (mtDNA) activates autoinflammatory dendritic cells (DCs) through the STING pathway [147]. However, deficiency of cGAS or STING has been reported to reduce the suppression of the TLR signaling pathway and fail to rescue TMPD-induced SLE, suggesting a more complex role of the cGAS-STING signaling pathway in SLE [148,149].

Notably, there is an obvious gender bias in the occurrence of SLE that reaches a male-to-female ratio of 1:9 [150,151]. Females also exhibit higher production of type I IFNs than males [150]. As IFNs regulate protein, STING expression is elevated in response to the high level of IFN-α, which is in turn amplified through the positive feedback loop between IFNs and estrogen receptor-α (ERα) [150]. Moreover, the upstream of STING IFI16 protein is also under the regulation of sex hormones, indirectly affecting the function of STING [151,152]. These studies suggest that the cGAS-STING pathway might also participate in the gender bias observed in SLE. However, Congy–Jolivet et al. reported that cGAMP-induced IFN-α production was similar in both genders, while the sex bias of IFN-α depended on TLR7 [153]. In order to determine the role of STING in the SLE gender bias, more related research is required.

Infection is the primary death cause of SLE patients [154,155,156]. Glucocorticoids and immunosuppressants are the main drugs used to treat SLE. High-dose or long-term application of glucocorticoids increases the risk of immunosuppression and susceptibility to a variety of pathogens [157]. Belimumab is the first biologic approved by the US Food and Drug Administration (FDA) for the treatment of SLE [158]. It is a human monoclonal antibody that binds to and inactivates B-cell activating factor (BAFF), inducing apoptosis and inhibiting B-cell maturation [158] while reducing the number of anti-dsDNA antibodies and increasing complement C3 and C4 levels [159,160]. Although belimumab contributes to the reduction of glucocorticoid dose, its long-term toxicity cannot be ignored. Abatacept is a fusion protein between cytotoxic T-lymphocyte-associated protein 4 (CTLA-4) and human IgGI Fc segments, which inhibits T-cell activation by blocking the interaction between CD28 and CD80/CD86 [161]. It has been found to significantly reduce anti-dsDNA antibody levels and urinary protein/creatinine levels in patients with SLE [162,163]. Notably, IL-6 is implicated in B-cell differentiation and autoantibody production. IL-6 expression in renal tissue is rising in lupus nephritis patients [164]. Animal experiments have also shown that IL-6 deficiency can effectively delay the occurrence of lupus nephritis, improve kidney function, and prolong the survival of SLE mice [164]. Therefore, it is expected to treat SLE by properly inhibiting the STING pathway and downregulating IL-6 expression [25].

### 8.3. STING and Infectious Diseases

Most patients infected with bacteria suffer from pulmonary dysfunction, which is accompanied by cardiovascular instability and the deterioration of renal function [165]. Bacteria and viruses can stimulate excessive inflammatory responses through various pathways. Inflammatory cells can release diverse toxic mediators to destroy tissue structure, cause metabolic disorders, and even induce organ dysfunction [166]. In recent years, infectious diseases caused by viruses have had higher pathogenicity, infection rates, and mortality rates than other types of microbial infections due to the lack of effective treatment.

Although type I IFN often shields mammalian hosts from viral infections, it can be pathogenic in some cases [167,168,169,170,171]. Despite the decreased production of ISGs and inflammatory cytokine genes, type I IFN was found to prevent mice from a fatal illness at one day post-infection (before viral titers peaked). However, if delayed IFN-β treatment fails to suppress viral replication effectively, monocytes, macrophages, and neutrophils will infiltrate and expression of proinflammatory factors will increase in lung tissue, resulting in fatal pneumonia or sublethal infection [169,172]. In addition to the rapid replication of the virus, a delay in the IFNs signaling pathway also appears in the coronavirus, promoting the accumulation of pathogenic inflammatory mononuclear macrophages and leading to increased levels of pulmonary cytokines/chemokines [169,171,173]. Autopsy of patients with multiple organ failure revealed massive neutrophil infiltration in renal vessels and lung tissues [174,175,176,177]. In non-lung tissues, neutrophils bind tightly to endothelial cells in postcapillary venules, resulting in vascular occlusion, tissue hypoxia, and hypoperfusion, while neutrophils release lytic factors that increase vascular permeability [166]. In lung tissue, when neutrophils are activated, extracellularly released proteolytic enzymes and oxygen radicals can directly lead to tissue damage [166]. The survival of higher species depends upon the proper control of innate immunity, as pathogens proliferate in the absence of inflammation and cause tissue damage when inflammation is severe [178,179].

The most common type of pneumonia is bacterial pneumonia, and 80% of children’s pneumonia is caused by bacteria [180]. *Klebsiella pneumoniae* is the most common pathogen. Infection with *Legionella pneumophila* is considered to be a significant contributor to inpatients and outpatients developing pneumonia. This pneumonia is often called Legionnaires’ disease, and despite effective antibiotic treatment, its mortality rate is still about 8~34% [181]. When *Klebsiella pneumoniae* invades the lower respiratory tract, it can cause congestive edema of the alveolar capillaries, infiltration of inflammatory cells into the alveoli, and exudation of red blood cells [182]. Mitochondria were found to be the core factor determining the destiny of dying cells [183,184]. When caspases are inactive, dying cells induce necrosis by releasing mtDNA, which in turn triggers the production of numerous inflammatory factors via STING activation. When caspases are active, apoptotic proteases in mitochondria can be activated spontaneously in order to inhibit the inflammatory response caused by DAMPs (especially mtDNA), and cells progress to apoptosis [183,184]. The structure of mtDNA is similar to that of bacterial DNA [185]. Under normal physiological conditions, mtDNA is in the mitochondria, and under pathological conditions, it is released into the cytoplasm [185]. H_2_O_2_ produced by *Streptococcus pneumoniae* stimulates type I IFN secretion depending on the activation of the STING pathway, causing mitochondrial damage in host cells and destroying the defense function of the organism [186,187,188]. The dengue virus’ C-terminal mitochondrial membrane is targeted by M protein, which results in swelling, permeabilization, and loss of the membrane [189,190]. Cytoplasmic translocation of mtDNA can induce cGAS- and DDX41-dependent innate immune responses during infection with cerebral myocarditis virus or influenza virus [191]. In addition, antiviral signals dependent on the STING pathway can be amplified in neighboring cells through gap cell junctions [192]. Some viruses, such as the hepatitis B virus (HBV), hepatitis C virus (HCV), human immunodeficiency virus (HIV), classical swine fever virus (CSFV), and porcine reproductive and respiratory syndrome virus (PRRSV), can promote mitochondrial division and induce mitochondrial autophagy as a means of maintaining persistent infection and attenuating the innate immune response [168,193,194,195,196]. In addition to viral-induced infectious pneumonia, increased dsDNA and C-X-C motif chemokine ligand 10 (CXCL10) have been detected in the sputum of silicosis patients [197]. STING activation and elevated CXCL10 expression have also been found in the lung tissue of patients with interstitial lung fibrosis [197]. Moreover, the STING-PERK-eIF2α pathway was reported to contribute to organ fibrosis, and pharmacological or genetical inhibition of this pathway can attenuate lung fibrosis [56].

Viral myocarditis is mainly caused by enteroviruses and upper respiratory tract viruses, both of which can damage myocardial tissue directly or through the immune system [198,199,200,201]. During the early stages of viral infection, NK cells and macrophages infiltrate into the tissues and play an early antiviral role [199].Then, during the middle stages of infection, T cells infiltrate these tissues, and IL-17 increases viral replication and inflammatory response [200,201]. Specific anti-myocardial antibodies produced by antigen mimicry will aggravate myocardial damage [199,200,201]. The STING signaling pathway activated by viral nucleic acids is not only able to activate the immune system, but also to participate in autophagy. Coxsackievirus RNA replication requires autophagy in order to form autophagosome-like bilayer membrane vesicle-folding structures, and inhibition of autophagy can block the replication of the Coxsackievirus [202].

Bronchial asthma is a heterogeneous disease with chronic airway inflammation as its primary characteristic. This chronic condition can result in reversible airway damage in its early stages and irreversible damage in later stages. Although asthma remains a difficult disease to cure, the symptoms of most asthma patients can be managed through proper strategies. The causative factors of asthma are genetic, infectious, and environmental. Rhinovirus, respiratory syncytial virus, Mycoplasma pneumoniae, and Chlamydia are the most common pathogens causing the onset, development, and acute deterioration of asthma clinically [203,204]. Frequent acute attacks can lead to decreased lung function and even death. Most studies in vitro show that the synthesis of IFNs is insufficient in asthmatic patients after viral infection [205]. In contrast, after rhinovirus infection and acute attack, the level of IFNs in the nasopharyngeal fluid of patients is significantly increased, and the severity of the acute attack is positively correlated with the level of IFNs clinically [206,207]. Thus, inadequate IFN synthesis may occur at the initial stage of viral infection [207]. After viral replication, IFN synthesis triggers an inflammatory cascade response that exacerbates asthma attacks. Indeed, IL-4, IFNs, and IgE were increased in the alveolar lavage fluid of asthmatic patients [208]. After invading the respiratory tract, Mycoplasma pneumoniae adheres to the respiratory epithelial cells, absorbs their nutrients, and produces metabolites such as oxygen radicals that damage the epithelial cells [209]. Epithelial cells can release multiple inflammatory factors and mucin after injury [210]. Pulmonary macrophages clear Mycoplasma pneumoniae through the MyD88-NF-κB pathway [211], while Mycoplasma pneumoniae induces a strong inflammatory response within macrophages through autophagy and TLR-4 [212]. Therefore, inhibition of the intense inflammatory response can alleviate an acute asthma attack, and this may be achieved by inhibiting the STING pathway.

The relationship between STING and SARS-CoV-2 is complex [213]. During the initial stages of COVID-19 infection, the virus effectively suppresses STING activation by directly binding viral accessory protein ORF3a to STING [214]. This suppression of the natural immune response provides the virus with an opportunity to proliferate [214]. As the infection progresses, the amount of virus in the host body gradually increases, and the virus fuses with host cells to form syncytia [215]. Due to the genomic instability, the micronucleus in the syncytia activates the STING pathway in order to mediate the inflammatory response [216]. Therefore, STING agonists or inhibitors show potential for the treatment and prevention of the SARS-CoV-2 virus. Specifically, STING agonists are utilized early in the infection to activate the host’s innate immune response and curb the virus’s replication. STING inhibitors are further used later in order to attenuate excessive immune responses and reduce lung inflammation, thereby mitigating tissue damage. Further research is required in order to fully comprehend the interaction between SARS-CoV-2 and the STING pathway as an RNA virus.

### 8.4. STING and SAVI

SAVI is a kind of autoimmune disease induced by STING mutations with characteristics including early-onset systemic inflammation, interstitial lung disease, and severe skin vascular disease of the acellular region [217,218]. In 2014, Liu et al. identified the N154S, V155M, and V147L mutations in STING1 which can induce STING activation without cGAMP, thereby releasing type I IFNs [219]. As research has progressed, more SAVI-related mutations in STING have been found, including V155M, G166E, C206Y, G207E, R281Q, R284G, R284S, and H72N [66]. Mutations located on the spiral ring of the connector, including N154S, V155M, and V147L, lead to a 180° rotation of STING LBD, resulting in STING oligomerization and activation without cGAMP [220]. Mutations located at the polymerization interface, including C206, R281, and R284, can activate STING by relieving the auto-inhibition of STING oligomerization [221].

Currently, the primary clinical treatment used for SAVI is JAK inhibitors [218]. JAK inhibitors can inhibit the phosphorylation of STAT1 and thereby suppress the type I IFN pathway while affecting IL-6 and TNF-β secretion. However, SAVI patients treated with JAK inhibitors are at risk of viral respiratory infection [222]. The JAK1/2 inhibitor ruxolitinib produced a poor therapeutic effect in R281Q patients [218]. Furthermore, the SAVI phenotype may develop independently of type I IFN signaling, as evidenced by the fact that knockout of the type I IFN receptor or IRF3 in STING N153S/WT mice was reported to fail to alleviate lung disease [223,224]. As the understanding of STING continues to evolve, the potential value of STING inhibitors as a treatment for SAVI has become increasingly apparent. Studies have found that STING inhibitor NO_2_-FA can reduce type I IFN expression in fibroblasts with STING mutations, including V174L, N154S, and V155M [102]. Another STING inhibitor, SN-011, has been reported to abrogate the activation of the SAVI-associated mutant of STING [106]. Therefore, these findings suggest that STING inhibitors may represent a promising avenue for treating SAVI in the future.

### 8.5. STING and CNS Diseases

While STING is ubiquitous in most human tissues, STING cannot be detected in brain cells. However, studies have found that neuronal cells infected with the Japanese encephalitis virus (JEV), an RNA virus, could detect the expression of STING through immunohistochemistry [225]. Traumatic brain injury (TBI) often induces long-term neurological and psychiatric changes in patients with brain injury. When the central nervous system (CNS) is injured, the molecular patterns associated with the injury, released from the damaged neurons, activate glial cells that drive secondary neuroinflammation, such as cytosolic and mtDNA [226,227]. STING expression in the brain was increased in TBI patients 6 h after injury, and knocking out the STING gene in TBI mice can significantly inhibit the activation of astrocytes and the release of proinflammatory factors, elevate the levels of autophagic markers including LC3 and p62, and reduce the volume of brain damage [228]. Meanwhile, elevated expression of IFN and related genes has been reported in early and persistent TBI [229], so inhibiting the production of IFN-β through gene knockout or drugs can reduce post-traumatic neuroinflammation and neurodegeneration [229].

Amyotrophic lateral sclerosis (ALS), also known as acromegaly, is an incurable and fatal neurodegenerative disease. In a TAR DNA binding protein-43 (TDP-43)-induced ALS model, NF-κB induced increased levels of proinflammatory factors and IFNs [230]. Deficiency of STING reverses dopaminergic neuron deficits and motor deficits in the substantia nigra par compacta [231]. STING inhibitor H-151 has been reported to reduce the expression of type I IFN and alleviate neurodegeneration both in vitro and in vivo [230].

Pain is also associated with STING. Pain assists host defenses by alerting organisms to potentially damaging stimuli, including pathogens and cancer cells, through the use of peripheral pain-sensing neurons [232,233,234]. The expression of STING is high in nociceptive receptors [234]. Mice lacking the STING gene or type I IFN are hypersensitive to nociception and have enhanced nociceptive excitability [234]. In contrast, intrathecal STING activation in mice or non-human primates produces strong analgesic effects [234]. The role of STING in pain perception is regulated by the type I IFN pathway. Therefore, it may be helpful in the treatment of chronic pain or congenital analgesia by activating or inhibiting the STING pathway.

### 8.6. STING and Inflammatory Bowel Disease

Inflammatory bowel disease (IBD) is a type of archetypical inflammatory disease [235], and STING activation has been linked to the development of IBD [236]. STING-deficient mice suffer less from severe colitis, while STING agonist exacerbates dextran sulfate sodium-induced colonic damage and inflammation [237]. In addition, atrial natriuretic peptide is reported to repair the gut barrier through the inhibition of STING pathway phosphorylation in epithelial cells [238]. However, the effects of STING remain controversial [239]. Canesso et al. have reported that STING^-/-^ mice have defective intestinal mucosal protection mechanisms and are more susceptible to intestinal inflammation than WT mice [240]. Additionally, STING can upregulate the expression level of antimicrobial peptide in epithelial cells, thereby alleviating intestinal inflammation [241]. Above all, STING may be important for maintaining intestinal homeostasis and controlling intestinal inflammation, presenting the possibility of using STING inhibitors to treat IBD.

### 8.7. STING and NAFLD

Nonalcoholic steatohepatitis (NASH) is a lesion stage in NAFLD. At present, there is no clinically effective drug for the treatment of NASH. The “second strike” is considered the primary pathogenic mechanism of NASH; during this event, insulin resistance and excess free fatty acids in hepatocytes induce oxidative stress and hepatotoxicity [242]. Subsequently, inflammatory responses and oxidative stress lead to inflammatory liver injury and fibrosis [242]. STING protein has been reported to be deficient in human and mouse hepatocytes [20]. Interestingly, some studies found that STING was expressed in mouse hepatocytes associated with its pro-apoptotic effect [243,244]. In 2016, Arvin et al. found that the acute CCl_4_ modeling was able to promote hepatocyte apoptosis and liver fiber development via the STING-IRF3 pathway [243]. However, the IRF3 apoptosis promotion was independent of its function in inducing type I IFNs [243]. When STING or IRF3 is knocked down, inflammatory factor expression is reduced, glycogen stores are increased, and lipid accumulation and fibrosis are diminished [244,245]. Luo et al. demonstrated that human hepatocytes do not express STING and that STING is expressed in liver nonparenchymal cells, mainly Kupffer cells [245]. Compared to healthy individuals, STING expression is increased in the liver tissue of patients with NAFLD [246]. Hepatic stellate cells (HSCs) are the primary cells involved in liver fibrosis [246]. STING can enhance the activation of HSCs directly or through the macrophage paracrine pathway [247]. The STING-PERK-eIF2α signaling pathway has been shown to drive organ fibrosis, and this fibrosis can be attenuated by the depletion of STING or PERK [56]. The correlation of the STING pathway with apoptosis, inflammation, and fibrosis suggests that STING inhibitors have the clinical potential to prevent or cure NAFLD.

### 8.8. STING and Diabetic Complications

Diabetes can cause multiple complications, including cardiovascular diseases and organ failure [248]. Diabetic cardiomyopathy (DCM) is one of the major causes of death in diabetic patients, and there is no specific treatment strategy [249]. mtDNA-induced activation of the cGAS-STING pathway has been found to promote cardiac pyroptosis and hypertrophy via a nucleotide-binding oligomerization domain-like receptor pyrin domain containing 3 (NLRP3) in an inflammasome-dependent manner [249]. Knockdown or inhibition of STING can ameliorate cardiac hypertrophy and reduce the inflammation response in DCM mice [23,249]. The meteorin-like hormone can inhibit the cGAS-STING pathway through autophagy and alleviate diabetic cardiomyopathy [250].

Studies have also demonstrated that STING contributes to the development of diabetic kidney disease [251]. Cytoplasmic leakage of mtDNA was sufficient to cause kidney inflammation via activation of the cGAS-STING signaling pathway [252,253]. Knockdown or pharmacological inhibition of STING can reduce podocyte injury and improve renal functions in mouse diabetic models [253]. In addition, Feng et al. found that STING expression was upregulated at diabetic skin wound sites, where STING activation delayed wound healing [254], and silencing or inhibiting STING accelerated wound healing [254]. These findings suggested that STING inhibitors have promising potential for the treatment of diabetic complications.

### 8.9. STING and Other Diseases

Aicardi–Goutières syndrome is a type of autosomal recessive disorder induced by DNA abnormal degradation, and it presents neurological and cutaneous involvement as its main pathological features. It occurs due to gene mutations that encode multiple proteins, including exonuclease 1 (TREX1) and ribonucleic acid. Mutations in RNASE H2 are common [255,256]. TREX1-encoded proteins are involved in the metabolism of ssDNA and dsDNA. Widely expressed in cells, RNASE H2 functions in the degradation of RNA–DNA heterologous complexes as well as ribonucleotide excision and repair [255,256]. DNA nucleases are crucial for the maintenance of immune tolerance and the recognition and removal of DNA from foreign pathogens. Mutations in the genes encoding DNA nucleases can lead to impaired immune tolerance and abnormal accumulation of DNA degradation products, ultimately inducing autoimmunity [257].

As a specific autoimmune disease, rheumatoid arthritis (RA) is characterized by persistent inflammation and joint destruction. The migration and invasion of fibroblast-like synovial cells (FLS) enhances the joint inflammation and cartilage destruction of RA [258,259]. cGAS expression was elevated in RA-FLS, promoting their proliferation and enhancing TNF-α-mediated inflammation [259]. Likewise, the accumulation of dsDNA contributed to FLS-mediated rheumatoid arthritis synovial inflammation, which can be alleviated by the knockdown of cGAS or STING [260,261]. Additionally, TNF also results in joint swelling by inducing IFN response through the cGAS-STING pathway [262].

Myocardial infarction leads to cardiomyocyte death, dsDNA release, and STING activation, promoting IFN-β over-production [263]. IFNs diffuses through the local microenvironment and acts on IFNAR-expressing cells in an autocrine and paracrine manner, driving an increase in inflammatory factors and the infiltration of immune cells into myocardial tissue, in turn leading to myocardial tissue damage and impaired cardiac function [263]. Therefore, if the IFN-dependent inflammatory response due to cell death can be temporarily suppressed, it could reduce the incidence of heart failure caused by adverse ventricular remodeling.

Senescent cells can secrete proinflammatory cytokines (including IL-6, IL-8, and IL-1β) under the regulation of the NF-κB pathway which are known as senescence-associated secretory phenotypes (SASP) [80,264]. During cellular senescence, the DNA-damage response is a significant process [265]. The leakage of mtDNA and nuclear DNA can be recognized by DNA-sensing proteins like cGAS and IFI16, resulting in the activation of the STING pathway and SASP [266,267,268]. Furthermore, the senescent fibroblasts exhibit increased STING expression [269]. The knockout and inhibition of STING alleviate senescence and suppress SASP [56,270]. Interestingly, STING R293Q mutant, with a mild deficiency in IFNs-induction, is linked to a decreased risk of aging-related diseases [64,271,272]. In addition, the cGAS-STING-PERK pathway is also pivotal for DNA-damage-triggered senescence upon the STING-mediated translation reprogramming [56]. Thus, STING might become a novel target to treat aging-related inflammation.

## 9. Discussion

Inadequate innate immune responses lead to massive pathogen replication and severe infections, whereas failure to properly regulate the immune system can cause unlimited damage challenges to human organs [178,179]. In response to multiple stimuli, inflammatory and autoimmune diseases can arise due to the body’s inability to adjust the immune response to normal levels. The cGAS-STING pathway is a crucial mechanism in the cellular sensing of DNA and the activation of innate immunity, and it is implicated in various infectious and autoimmune diseases, such as psoriasis and SLE. Nevertheless, the existing treatment strategies for STING-related diseases have limitations. For example, current psoriasis treatments can generally only alleviate clinical symptoms, and they prolong the remission periods [273]. The high cost of biological drugs for psoriasis adds to the burden of patients, and this disease is prone to relapse after drug withdrawal [274]. While short-term efficacy is good, the risk of long-term exacerbation and side effects increases [136]. Therefore, the utilization of biological drugs clinically requires a strict selection of symptoms and disease course. Small-molecule therapy has dominated the treatment mode of modern medicine for a long time. Although its specificity is relatively lower than that of biological agents, it can be modified to bind to specific targets through various mechanisms [275]. Similarly, patients with SLE infection relying on glucocorticoids and monoclonal antibodies are also plagued by high costs and side effects [159,160]. For these diseases, small-molecule STING inhibitors may become a promising high-quality and low-cost treatment. By inhibiting STING, excessive inflammatory response and damage can be partially reduced, which slows down the progression of the disease and prevents the development of complications. In particular, SAVI induced by STING mutants may be rescued completely by targeting STING instead of IFNs. Moreover, STING inhibitors have shown therapeutic effects in relevant mouse models, including those of psoriasis and diabetic complications [138,254].

Compared to the use of mature STING activators as anti-tumor immunotherapy, research on STING inhibitors is still in early stages. STING inhibitors reported upon so far have demonstrated limitations, including low potency or poor drug-like properties. The current challenge facing STING inhibitors is to improve their bioactivity and physicochemical properties through structural modification. Researchers must also consider the safety of STING inhibitors and assess their potential side effects. First, patients with chronic inflammatory and autoimmune diseases usually require long-term medication, making it critical to establish a high safety window for the clinical application of STING inhibitors. Second, STING inhibitors functioning as immunosuppressants might carry the risks of disrupting the innate immune homeostasis of normal organs and thereby inducing infection, as the STING^−/−^ mouse model demonstrates [240]. However, pharmacological studies related to STING inhibitors, especially their toxicity and potential side effects, are still lacking. Additionally, due to the high heterogeneity of hSTING genes, the efficacy and response rates to a single STING inhibitor may vary among carriers of different STING genotypes. These challenges suggest that the development and optimization of STING inhibitors requires improving potency, drug-like properties, and specificity and finding proper bioactivity in order to avoid the side effects caused by over-inhibition. Taken together, safe and effective STING inhibitors hold great promise as novel therapeutic approaches to a variety of acute and chronic diseases.

## Figures and Tables

**Table 1:** An overview of STING inhibitors.

Inhibitor [Ref]	Binding Sites	Molecular Mechanism	Biological Activity
Nitro-fatty acid Derivatives [102]	C88, C91 at palmitoylation site and H16 in N-terminus	Covalently bind to the STING cysteines residues, block STING palmitoylation and inhibit STING activation	N.D.
C-176/178/170 and H-151 [103]	C91 at palmitoylation site		IC_50_ (H-151) = 134.4 nM (HFFs cells)
BPK-21/25 [104]	C91 at palmitoylation site		ISRE-Luc activity (BPK-25) = 3.2 μM (THP1 cells)
Astin C [21]	CDN binding site	Compete with cGAMP for the CDNs binding pocket and inhibit STING activation	IC_50_ = 3.42 ± 0.13 μM (MEFs cells)
Compound **18** [105]	CDN binding site		IC_50_ = 68 nM (STING^HAQ^); IC_50_ = 11 μM (THP1 cells)
SN-011 [106]	CDN binding site		IC_50_ = 502.8 nM (HFFs cells)
Palbociclib [107]	CDN binding site	Interact with STING CTD and block STING dimerization	IC_50_ = 0.81 ± 0.93 μM (HEK293 cells)
Compound **30** [108]	CDN binding site	Undetermined	IC_50_ = 1.15 μM (RAW264.7 cells)
6,5-heterocyclic derivatives	Unknown	Undetermined	IC_50_ ranges from 30 μM to less than 10 nM
SP23 [109]	Palmitoylation site	Promote the degradation of STING via ubiquitin-proteasome pathway	DC_50_ = 3.2 μM (THP1 cells)

## Data Availability

Not applicable.

## Sample Availability

Samples of the compounds are not available from the authors.
